# Comparison of Aroma Profiles of Whiskeys Fermented from Different Grain Ingredients

**DOI:** 10.3390/foods13132031

**Published:** 2024-06-26

**Authors:** Siqian Guo, Dan Wang, Yanting Li, Jingming Li, Jinkun Du

**Affiliations:** 1 College of Food Science & Nutritional Engineering, China Agricultural University, No. 17 Tsinghua Dong Road, Haidian District, Beijing 100083, China; guosiqian2022@163.com (S.G.); xdbzwd@163.com (D.W.); lijingming@cau.edu.cn (J.L.); 2 Faculty of Food Science and Engineering, China Agricultural University-Sichuan Advanced Agricultural & Industrial Institute, Chengdu 611430, China; 3 College of Agronomy and Biotechnology, China Agricultural University, No. 2 Yuanmingyuan Xi Road, Haidian District, Beijing 100193, China; 18838999041@163.com

**Keywords:** whiskey, aromatic evaluation, barley, wheat, highland barley, sorghum

## Abstract

Different grain sources of whiskey have great potential for aroma expression. In this paper, four whiskeys fermented from different raw materials (barley, wheat, highland barley, and sorghum) were compared. Gas chromatography–mass spectrometry (GC-MS) and sensory evaluation were used to determine the composition of the aromatic compounds. A correlation analysis was further conducted between the aromatic compounds and sensory evaluations. Barley whiskey and wheat whiskey had more pronounced fruity, floral, and grain aromas, attributed to esters and terpenes. Barley whiskey had the most compounds (55), followed by highland barley whiskey (54). Highland barley whiskey had the greatest number of unique aroma compounds (seven). It exhibited a unique cocoa aroma related to concentrations of *trans*-2-nonenal, γ-nonanolactone, 1-nonanol, isoamyl lactate, 2-butanol, and 6-methyl-5-hepten-2-one. Sorghum whiskey had a specific leather and mushroom aroma attributed to 6-methyl-5-hepten-2-one, ethyl lactate, ethyl caprate, phenethyl octanoate, farnesol, α-terpineol, 3-methyl-1-pentanol, and methyleugenol. Alcohols were the main aroma components of grain whiskeys. Isoamyl alcohol (231.59~281.39 mg/L), phenylethyl alcohol (5.755~9.158 mg/L), citronellol (0.224~4.103 mg/L), β-damascenone (0.021~2.431 mg/L), geraniol (0.286~1.416 mg/L), isoamyl acetate (0.157~0.918 mg/L), phenylacetaldehyde (0.162~0.470 mg/L), linalool (0.024~0.148 mg/L), 1-octen-3-ol (0.016~0.145 mg/L), *trans*-2-nonenal (0.027~0.105 mg/L), and *trans*-2-octen-1-ol (0.011~0.054 mg/L) were all important aroma compounds in the whiskeys.

## 1. Introduction

There are numerous whiskey production regions globally, with the primary five being the Scottish, American, Canadian, Irish, and Japanese whiskey regions, distinguished by their yield and influence [1]. Variations in the raw materials used for whiskey production are determined by local conditions. Single malt whiskey is considered the most traditional form, while whiskeys made from grains such as corn, wheat, and rye also hold a significant market share in the global whiskey industry [1,2]. Different ingredients contribute to the unique flavor profile of whiskey. The flavor components of whiskey are primarily derived from metabolites produced during yeast fermentation under varying nutrient compositions. These include the secondary metabolites in the microbial metabolism process: pyridines, sulfides, lactones, esters, alcohols, terpenes, ketones, aldehydes, organic acids, and indoles [3,4,5]. The grain ingredients are the key factors in the nutrient composition. In addition, reactions in the whiskey-making process, such as the Maillard reaction, have a direct impact on the flavor of the whiskey [3].

Among single malt whiskeys, barley whiskey has been the most studied [1,6]. The starch content of barley ranges from 45% to 68% [7]. During fermentation, yeast converts starch into alcohol, which directly influences the sensory attributes and alcohol content of the end products [8]. Moreover, barley is rich in vitamin E, vitamin B, minerals, and phenolics, which contribute to the unique and authentic flavor [7,9]. A study found that (E)-2-nonenal (fried/toasted/fatty), β-damascenone (honey/tea/plum), 3-methyl-1-butanol (fermented/yeast), furfural (baked/toasted almond), and ethyl hexanoate (fruity/apple) were the most influential odorants contributing to the flavor of newly made spirits [6].

Wheat (*Triticum aestivum* L.) is a major world crop with a high carbohydrate content (75–81%) [10], and it is one of the most important ingredients in alcoholic drinks. It has been used to replace barley in the production of Irish whiskey [1]. In the Code of Federal Regulations, wheat whiskey must contain wheat raw material over 51% (https://www.ecfr.gov (accessed on 20 January 2024)). In the study of Morris et al., wheat as an ingredient improved Irish whiskey alcohol grades [11]. Wheat performed well in the volatile profiles among the agricultural distillates of different botanical origins (maize, wheat, triticale, rye) in the report of Biernacka and Wardencki [12].

Highland barley (*Hordeum vulgare* L. var. *nudum* Hook. f.) is known as Qingke and is mainly planted in Tibet, China; it has a high protein, dietary fiber, and vitamin content and a low sugar content [13,14]. Highland barley accounts for 98% of Tibetan barley production. Highland barley liquor is produced through fermentation of the hulled seed [15]. It has a mild flavor with a pure highland barley aroma and a high content of volatile compounds: ethyl acetate, ethyl 2-methyl propanoate, ethyl butanoate, ethyl 3-methyl butanoate, ethyl pentanoate, ethyl hexanoate, ethyl octanoate, 3-methylbutanal, 1-octen-3-ol, and β-damascenone [13,15]. Compared with barley, highland barley is a more accessible and cost-effective ingredient for brewing local Chinese whiskey [15].

Sorghum (*Sorghum bicolor* (L.) Moench) is an important ingredient in alcoholic beverages, including Chinese liquor, African sorghum beer, and Rwandan Urwagwa (a traditional Rwandan banana alcoholic beverage) [16,17,18]. Sorghum has a high starch content of 55–80%, excluding gluten, and is an ingredient in brewing whiskey [19]. Lopes et al. reported that sorghum is a good substrate for alcoholic fermentation to produce spirits, reducing brewing costs [20]. Compared with other grains, sorghum contains a large amount of tannin, which can increase the mellow aroma of wine, and it is considered a significant factor affecting the flavor of sorghum liquor [16]. A study has shown that among the five liquors (brewed from sorghum, wheat, corn, rice, and barley), sorghum liquor has the best flavor and is characterized by a high ester and low aldehyde content [16].

The effects of different brewing materials on the quality and flavor profile of whiskey are worth exploring. In particular, limited research has been conducted on Chinese local highland barley and sorghum in whiskey production. Comparative analysis reports on the flavor of whiskeys made from various grain ingredients have not been widely conducted. To explore the feasibility of producing fermented whiskey from Chinese native crops, four grain ingredients (barley, wheat, highland barley, and sorghum) were selected, to analyze and compare their respective aroma characteristics. This paper provides a foundation for the informed selection of raw materials in whiskey production.

## 2. Materials and Methods

### 2.1. Samples Preparation

The barley (*Hordeum* L.) was produced in Australia, wheat (Wanmai 38) in Anhui, China, highland barley (Qinglv 1) in Qinghai, China, and sorghum (Liangnuo 1) in Liaoning, China. Four clean grain ingredients with a complete appearance were each selected and crushed to powder using a pulverizer. Then, the grain powder and water (grain powder–water = 1:4) were put into an American triple cask with circulating spray for 1.5 h at 65 °C. Saccharification solution with a specific gravity of 1.060–1.070 was obtained. After cooling, the saccharification solution was transferred to the fermenter. Then, the commercial yeast CR1 (at a dosage of 8 g/10 L) was activated, inoculated, and fermented at 25 °C for 72 h. Fermentation was ended when the specific gravity stopped falling and the final reducing sugar content remained stable. Following this, the fermentation liquid was transferred to a pot still to begin the first distillation. At this stage, the alcohol content was distilled to 1%. The double distillation process was then initiated. Liquors with an alcohol content of 1–2% of the total alcohol content were chosen as the heads, and 500 mL of newly made whiskey was collected and stored at room temperature for use. These procedures were repeated three times. Prior to testing, the samples were diluted to 10% (*v*/*v*) alcohol with ultra-pure water.

### 2.2. Chemicals

Standards: 2-butanol (≥99%), 1-propanol (≥99.9%), hexanal (98%), isoamyl acetate (≥99%), d-limonene (97%), 2-methyl-1-butanol (≥99%), ethyl hexanoate (≥99%), styrene (≥99%), 4-methyl-1-pentanol (97%), 2-heptanol (98%), 3-methyl-1-pentanol (≥99%), ethyl heptanoate (99%), ethyl lactate (≥98%), nonanal (≥98%), 2-octanol (≥99.5%), ethyl caprylate (≥99%), 1-octen-3-ol (≥98%), 1-heptanol (≥99.5%), coriander heptenol (≥95%), 2-ethyl-1-hexanol (≥99%), 2-nonanol (99%), benzaldehyde (≥99%), linalool (≥97%), isoamyl lactate (98%), ethyl caprate(≥99%), 1-nonanol (≥98%), α-terpineol (≥96%), 1-decanol (≥98%), citronellol (≥95%), ethyl benzeneacetate (≥98%), geraniol (≥97%), nerol (≥97%), phenethyl acetate (99%), β-damascenone (95%), geranylacetone (96%), isoamyl decanoate (97%), ethyl 3-phenylpropionate (99%), phenylethyl alcohol (≥99%), *trans*-nerolidol (≥85%), γ-nonanolactone (≥98%), octanoic acid (≥98%), 2,4-di-*tert*-butylphenol (99%), dibutyl phthalate (99%), 2-methyl-1-propanol (≥99.5%), 1-butanol (≥99.5%), isoamyl alcohol (≥99%), 1-pentanol (≥99%), 1-hexanol (≥99.5%), furfural (99%), *trans*-2-nonenal (97%), 1-octanol (≥99%), ethyl laurate (≥98%), 1-dodecanol (≥98%), ethyl myristate (99%), decanoic acid (≥98%), ethyl palmitate (≥99%), farnesol (≥95%), and 4-methyl-2-pentanol (98%) were purchased from Sigma-Aldrich (St. Louis, MO, USA). All standards were prepared as standard solutions and stored at −4 °C.

C8-C21 normal paraffins, NaCl, methanol (suitable for HPLC), ethanol (suitable for HPLC), dichloromethane (HPLC Plus), and phosphate-citrate buffer (PBS) were purchased from Sigma-Aldrich (St. Louis, MO, USA). Edible alcohol was purchased from Xinheyang Alcohol (Mengzhou, China), AR2000 glycosidase from Diagnos Med srl (Shirley, NY, USA), and commercial yeast CR1 from Oenofrance (Magenta, France).

### 2.3. Aroma Analysis

#### 2.3.1. Free Volatile Detection

The free volatile detection used headspace solid-phase microextraction–mass spectrometry coupled with gas chromatography–mass spectrometry (HS-SPME-GC/MS) and SPME fiber assembly Divinylbenzene/Carboxen/Polydimethylsiloxane (DVB/CAR/PDMS, 50/30 μm, Supelco, St. Louis, MO, USA). The SPME fiber was placed into the injection port (250 °C) for 1 h before detection. Then, 5 mL of the sample was added to a 15 mL headspace vial containing 2 g NaCl and 10 μL internal standard (4-methyl-2-pentanol, 1044 mg/L ethanol). The vial was placed in a water bath at 40 °C for 30 min. It was then extracted for 30 min at 400 r/min. After extraction, the fiber was inserted into the injection port and analyzed in splitless mode for 8 min. Each sample was replicated three times.

Gas chromatography–mass spectrometry (GC-MS, Agilent 7890B-5977B, Agilent Technologies, Santa Clara, CA, USA) was performed using an INNOWAX column (60 m × 0.25 mm × 0.25 μm, Agilent Technologies, Santa Clara, CA, USA). The oven temperature program was as follows: it was initially set at 40 °C for 3 min and then increased to 230 °C at a rate of 5 °C/min, remaining constant for 15 min. The carrier gas was helium, flowing continuously at a rate of 1 mL/min. The interface temperature was consistently maintained at 250 °C, while the ion source temperature was precisely set to 230 °C. The collision energy was 70 eV, and the scanning range was 30–400 *m*/*z*.

For qualitative analysis, the retention index (RI) and ion fragment information of mass spectra were compared with the NIST 17 library. The RI was calculated from n-alkanes (C7–C21) and verified against the standards. The standard curve was used to quantify the aroma substances of the whiskey. For the aroma components without standards, 4-methyl-2-pentanol was chosen as the internal standard and quantified using the following formula:

(1) $$\mathrm{Ci}=\frac{\mathrm{Cs}\times \mathrm{Ai}}{\mathrm{As}}$$

In the formula, Ai is the peak area of the components to be measured, As is the peak area of the internal standard, Ci is the content of the components to be measured, and Cs is the content of the internal standard.

#### 2.3.2. Bound Volatile Detection

Clearnert PEP (Polar Enhanced Polymer, 200 mg, 6 mL, Agela Technologies, Tianjin, China) was first activated with 10 mL methanol and 10 mL ultra-pure water. Then, a 5 mL sample solution was added to the PEP and eluted with 5 mL ultra-pure water and 10 mL dichloromethane successively. The result was finally eluted with 20 mL methanol and collected in a 50 mL round-bottomed flask. The eluent was then steamed in a vacuum at 30 °C until dry using an RV10 vacuum rotary evaporator (IKA-Werke, Staufen, Freiburg, Germany). A total of 5 mL PBS (pH = 5) was added for re-dissolution and transferred to a 15 mL headspace vial. After that, 100 μL AR2000 glycosidase solution (100 mg/mL, 2M PBS) was added to the vial, the cap tightened, and the vial placed in a water bath at 40 °C for 16 h for enzymatic hydrolysis. Each sample was replicated three times. Bound volatiles were detected by means of HS-SPME-GC/MS using the same method as for free volatiles.

### 2.4. Sensory Evaluation

The sensory evaluation procedure was conducted following the guidelines of GB/T 15038-2006, “Analytical methods of wine and fruit wine” [21], and GB/T 10220-2012, “Sensory analysis—Methodology—General guidance” [22]. The sensory panel’s preliminary evaluation of the whiskey samples resulted in the selection of 22 aroma descriptors: mellow, apple, sweet fruit, pineapple, honey, rose, malt, citrus, grass, cream, spicy, caramel, lemon, grain, coconut, banana, leather, mushroom, bread, fat, nuts, and cocoa. A total of 25 assessors (6 males and 19 females) participated in the descriptive analysis; all were 20–25 years old and undergraduate or postgraduate students majoring in food-related fields.

All participants participated in the training, which included an introduction to the basic theory and methods of sensory evaluation and whiskey samples. The training covered the identification, classification, and intensity rating of whiskey aromas, as well as training in the familiarity, memory, and description of characteristic whiskey aromas. The training sessions lasted 45 min each, with a total duration of no less than 15 h. The 8 primary aroma components of whiskey were used in the training solution: 1-octen-3-ol, phenylethyl alcohol, isoamyl acetate, linalool, β-damascenone, nonanal, ethyl hexanoate, and isoamyl alcohol. These aroma components respectively correspond to the following descriptors: mushroom, rose, banana, floral, sweet, citrus, pineapple, and malt. The training solution was prepared using 10% edible alcohol, based on the concentration found in whiskey. Then, the assessors were familiarized with the typical aroma of whiskey. The Triangle Test was used to assess the quality of the assessments.

The trained and tested assessors participated in the descriptive analysis of various whiskey samples. Following the whiskey flavor wheel as a reference, the assessors were asked to establish aroma descriptors using partial whiskey samples. The assessors were instructed to collect aroma descriptors by sniffing and then selecting characteristic aroma descriptors based on their frequency of occurrence. Four whiskey samples were each placed in whiskey ISO standard cups (220 mL) and labeled with three random numbers. Assessors were asked to smell each sample in order and select the perceived aroma from the characteristic aroma descriptors. Finally, the frequency, intensity, and preference of the aroma descriptors in each whiskey sample were statistically analyzed.

### 2.5. Statistical Analysis

The GC-MS data were analyzed using Agilent Masshunter Qualitative Analysis 10.0.10305.0 (Agilent Technologies, Santa Clara, CA, USA) and MSD Chemstation Data Analysis Application 6.0.0.0 (Agilent Technologies, Santa Clara, CA, USA). Data statistics and significance analysis were performed using IBM SPSS Statistics 27.0.1 (IBM, Armonk, NY, USA). Pie charts, Venn diagrams, radar charts, heat maps, PCA charts, and the correlation analysis were analyzed and drawn using OriginPro 2022 SR1 (OriginLab, Northampton, MA, USA). The association network diagram was created using Gephi 0.10.0 (https://gephi.org (accessed on 23 October 2023)).

## 3. Results and Discussion

### 3.1. Analyses of Whiskey Aroma

**Figure 1:**
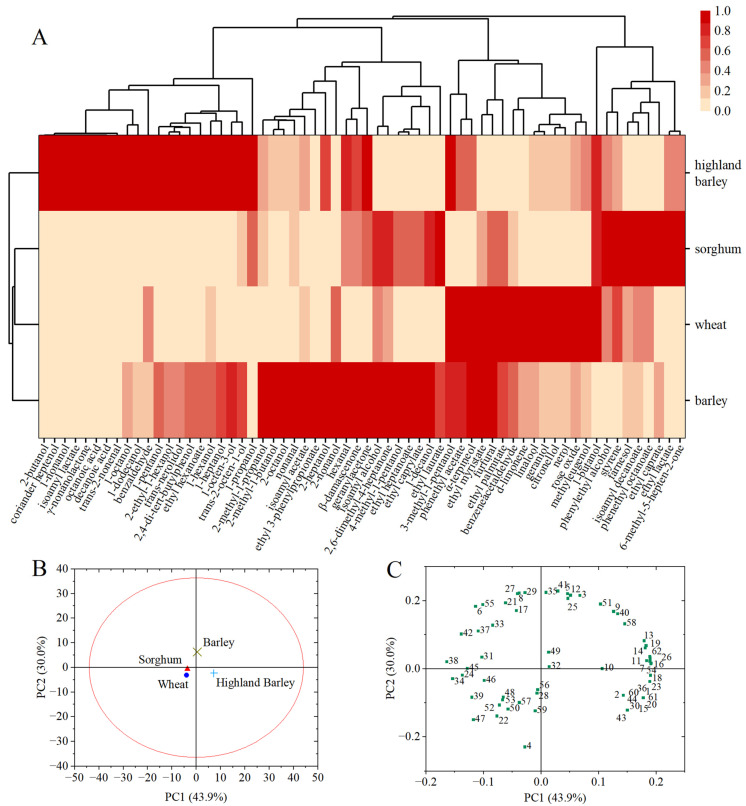
The heat map of the aroma composition content (**A**), PCA score plot (**B**), and loading plot (**C**) of the four whiskeys. The numbers in the loading plot correspond to the compound numbers in Table 1.

HS-SPME-GC/MS identified a total of 62 aroma compounds in all the whiskey samples (Table 1), including 24 alcohols, 15 esters, 6 aldehydes, 9 terpenes, and 8 other compounds. In terms of aroma components, barley whiskey and highland barley whiskey had the highest numbers of detected components, with 55 and 54, respectively. Wheat whiskey and sorghum whiskey had 45 and 46 detected aroma components, respectively. Figure 1A shows that the aroma components of barley whiskey were the most abundant and had the highest content, followed by those of highland barley whiskey. Alcohols were the major contributors to the aroma profiles of all four whiskeys.

**Figure 2:**
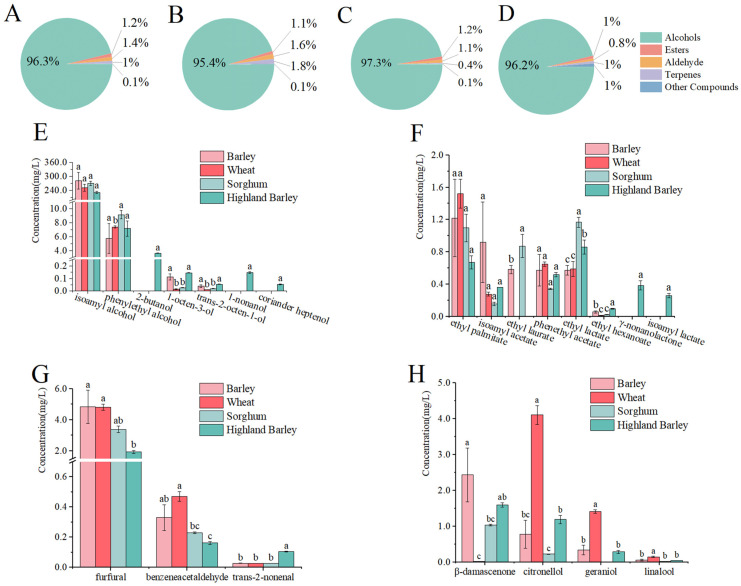
Aroma composition ratio of four whiskeys (**A**–**D**, barley whiskey, wheat whiskey, highland barley whiskey, and sorghum whiskey, respectively) and comparison of important aroma components (**E**–**H**, alcohols, esters, aldehydes, and terpenes, respectively). Different letters indicate statistically significant differences (*p* < 0.05).

According to Figure 2A–D and Table 1, alcohols were the most abundant aroma components in whiskey, accounting for over 95% of the total. These alcohols contribute fruity, floral, green, and fatty aromas to the sensory characteristics of whiskey. A total of 24 alcohols were detected in four whiskeys (21 in barley whiskey, 20 in highland barley whiskey, 16 in wheat whiskey, and 16 in sorghum whiskey), and 13 were common to all four whiskeys (Table 1). In terms of alcohol content, barley whiskey (373.454 mg/L) was the highest (wheat whiskey 326.346 mg/L, sorghum whiskey 353.392 mg/L, and highland barley whiskey 325.837 mg/L). Isoamyl alcohol accounted for more than 70% of the total alcohol content (Figure 2E), ranging from 231.586 to 281.389 mg/L. Isoamyl alcohol can provide a fruity and whiskey-like aroma to whiskey (Table 1). In addition, 1-octen-3-ol, *trans*-2-octen-1-ol, phenylethyl alcohol, and 1-nonanol were also primary alcohols in whiskey (OAV > 1). The concentrations of 1-octen-3-ol in highland barley whiskey (0.145 mg/L) and barley whiskey (0.113 mg/L) were significantly higher than those in wheat whiskey (0.015 mg/L) and sorghum whiskey (0.027 mg/L) (*p* < 0.05). The content of 1-octene-3-ol in the whiskeys was noticeably low. Nevertheless, owing to its low aroma threshold (0.006 mg/L), 1-octene-3-ol significantly contributed to the whiskey aroma, imparting earthy, green, and oily notes. 2-Butanol, 1-nonanol, and coriander heptenol were found only in the highland barley whiskey. 1-Nonanol was an important aroma component in highland barley whiskey (OAV > 1), with a fatty, floral, and orange aroma. 2-Butanol exhibited a fruity and sweet aroma, and coriander heptenol exhibited a sweet, oily, and green aroma, but the contents of both were below the threshold (5.000 and 2.000 mg/L).

Esters were the second largest group of compounds, giving the whiskeys fruity, floral, and sweet aromas (Table 1). A total of 15 esters were detected in the four kinds of whiskey (13 in barley whiskey, 12 in highland barley whiskey, 12 in sorghum whiskey, and 10 in wheat whiskey), 10 of which were common to all four whiskeys (Table 1). Among them, ethyl esters were considered the essential aroma components [23,24]. They accounted for the highest proportion, ranging from 70.1% (highland barley whiskey) to 95.1% (sorghum whiskey). Some high concentrations of the components ethyl palmitate, ethyl lactate, ethyl laurate, isoamyl acetate, and phenethyl acetate contributed to a strong fruity, sweet, and floral aroma. Ethyl palmitate, ethyl lactate, isoamyl acetate, and phenethyl acetate were the common aroma components in all four whiskeys. The concentration of ethyl lactate in sorghum whiskey (1.168 mg/L) was significantly higher than that in other whiskeys (*p* < 0.05). Ethyl laurate was detected only in barley whiskey (0.583 mg/L) and sorghum whiskey (0.874 mg/L). There was no significant difference in the concentrations of ethyl palmitate, isoamyl acetate, and phenethyl acetate among the four whiskeys (*p* > 0.05). Ethyl hexanoate, producing a pineapple and fruity aroma, was the common aroma component in all four whiskeys. In particular, highland barley whiskey had the highest concentration of ethyl hexanoate (0.096 mg/L). γ-nonanolactone, with a creamy and coconut aroma, and isoamyl lactate, with a fruit, cream, and nutty aroma, were detected only in highland barley whiskey. Due to its low threshold of 0.010 mg/L, γ-nonanolactone significantly contributed to the aroma of highland barley whiskey (OAV = 39.7).

Aldehydes were mainly derived from raw materials and the fermentation process [25]. Among aldehydes, furfural had the highest content (1.927–4.833 mg/L), exhibiting a bread, woody, and sweet aroma. In highland barley whiskey, the level of furfural was significantly lower compared to that in other whiskeys (*p* < 0.05). The odor threshold of *trans*-2-nonenal was very low at 0.0006 mg/L, which greatly contributed to the fatty and grass aroma. The content of *trans*-2-nonenal in highland barley whiskey (0.105 mg/L) was significantly higher than that in other whiskeys (*p* < 0.05). Phenylacetaldehyde was also an important component in the flavor of whiskey (OAV > 1), exhibiting a green, sweet, and floral aroma.

Terpenes are generally considered to come from brewing ingredients, and most have a pleasant aroma and low threshold, making them a significant contributor to the aroma of whiskey [25,26]. Table 1 shows that the content of terpenes varied considerably in the whiskeys made from different ingredients. Wheat whiskey had the highest citronellol content (4.103 mg/L), with a rose and sweet aroma, accounting for 66.6% of the total terpenes. The concentration of β-damascenone was higher in barley whiskey (2.431 mg/L) and highland barley whiskey (1.592 mg/L). Due to its extremely low threshold (0.00012 mg/L), it played a significant role in aroma, contributing to the rose, apple, and honey notes. Geraniol and linalool were also prominent terpene aroma components (OAV > 1), and their contents in wheat whiskey were 1.416 and 0.148 mg/L, respectively. Geraniol contributed a floral, sweet, and fruity aroma, and linalool produced a citrus, floral, and sweet aroma. Furthermore, compared to the other three whiskeys, sorghum whiskey did not have a significantly higher content of terpenes and had the fewest varieties, with only seven.

On the whole, the OAVs of isoamyl alcohol (231.59~281.39 mg/L), phenylethyl alcohol (5.755~9.158 mg/L), citronellol (0.224~4.103 mg/L), β-damascenone (0.021~2.431 mg/L), geraniol (0.286~1.416 mg/L), isoamyl acetate (0.157~0.918 mg/L), phenylacetaldehyde (0.162~0.470 mg/L), linalool (0.024~0.148 mg/L), 1-octen-3-ol (0.016~0.145 mg/L), *trans*-2-nonenal (0.027~0.105 mg/L), and *trans*-2-octen-1-ol (0.011~0.054 mg/L) were all higher than 1, which demonstrated the significance of these aroma compounds in the whiskeys. The cluster analysis results (Figure 1A) and PCA analysis (Figure 1B) showed that the sorghum whiskey and wheat whiskey were similar, with the sorghum whiskey being rich in esters and the wheat whiskey being rich in terpenes. In addition, the highland barley whiskey had the largest number of unique aroma compounds, including 2-butanol, coriander heptenol, 1-nonanol, isoamyl lactate, γ-nonanolactone, octanoic acid, and decanoic acid (Table 1). The PCA analysis results (Figure 1B,C) indicated that the presence of principal component 2 distinguished barley whiskey from the other three. This difference was mainly due to the high content of esters such as ethyl caprylate (**29**), ethyl 3-phenylpropionate (**35**), and ethyl heptanoate (**27**), and lower levels of 1-butanol (**4**) in barley whiskey. Principal component 1 distinguished the highland barley whiskey from the other three, and this difference was mainly due to the presence of alcohols such as 2-ethyl-1-hexanol (**16**), 1-pentanol (**7**), 1-octanol (**18**), and 1-dodecanol (**23**) and esters such as ethyl palmitate (**38**), isoamyl decanoate (**34**), and ethyl hexanoate (**26**), and *trans*-nerolidol (**54**).

### 3.2. Analyses of Sensory Evaluation

A total of 25 trained assessors participated in the sensory evaluation. The panel screened the aroma descriptors of the whiskey and identified a final set of 22 descriptors: mellow, apple, sweet fruit, pineapple, honey, rose, malt, citrus, grass, cream, spicy, caramel, lemon, grain, coconut, banana, leather, mushroom, bread, fat, nuts, and cocoa.

**Figure 3:**
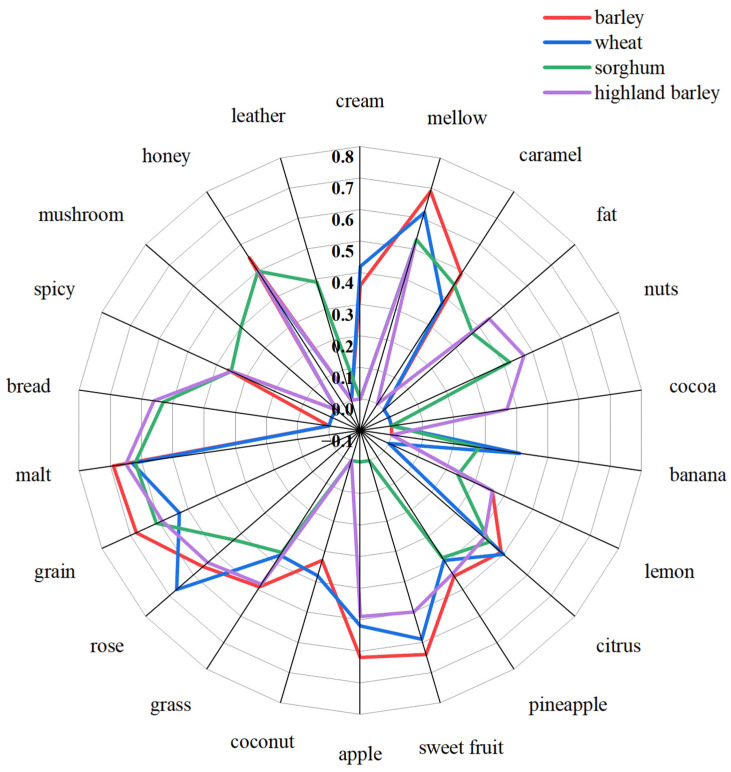
Flavor radar plots for the four whiskeys.

Figure 3 illustrates that mellow, citrus, pineapple, grass, rose, malt, grain, and honey aromas were outstanding among the four whiskeys. Barley and wheat are the traditional ingredients used in whiskey brewing, and the sensory scores for whiskeys made from these two ingredients were similar, with the most prominent flavors being fruity (citrus, pineapple, sweet fruit, apple), floral (coconut, grass, rose), and grain (malt and grain). These flavors were consistent with the classic outstanding aromas of whiskey, which are fruity, sweet, pungent, cereal/grainy, and woody [6,25]. Compared to barley whiskey and wheat whiskey, sorghum whiskey and highland barley whiskey received similar scores in seven sensory attributes: pineapple, citrus, grass, malt, grain, honey and mellow, while the spicy and bread aromas scored higher. Malt, grain, bread, mellow, and honey aromas were more prominent in sorghum whiskey (scores of >0.5). Furthermore, the sorghum whiskey had a unique leather and mushroom aroma, but the flower and fruit aromas (sweet fruit, apple, and coconut) were noticeably absent. Malt, grain, mellow, apple, sweet fruit, honey, rose, and bread aromas were more pronounced in highland barley whiskey (scores of >0.5). Highland barley whiskey had a cocoa flavor that was absent from the other whiskeys.

### 3.3. Correlation Analysis between Aroma Components and Sensory Evaluation

#### 3.3.1. Potential Interaction of Aroma Sensory Attributes

**Figure 4:**
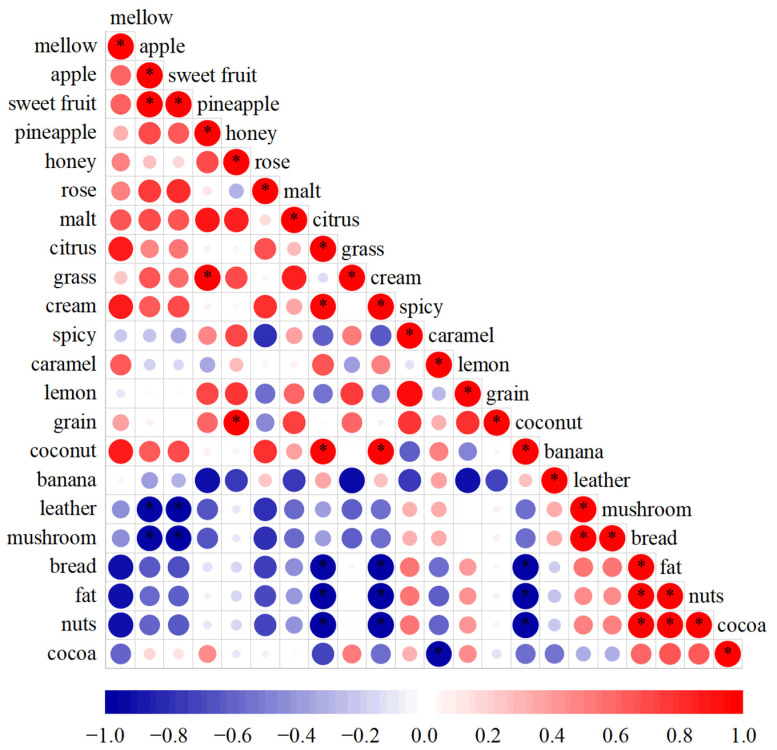
Scatter plot of correlation coefficients between aroma sensory attributes. The size of the circle corresponds to the correlation coefficient, and the color represents the positive/negative correlation (red = positive correlation, blue = negative correlation). * indicates a significant association at *p*-value = 0.05.

Figure 4 shows a significant positive correlation among the three sensory attributes (nuts, bread, and fat) (*p* < 0.05), and Boothroyd et al. also found a significant positive correlation between nuts and fat aromas [27]. Similarly, there were significant positive correlations between mushroom and leather; citrus, cream, and coconut; pineapple and grass; and sweet fruit and apple. The following negative correlations were also important, suggesting a possible masking effect among aroma sensory attributes, such as apple/sweet fruit to leather/mushroom, banana to pineapple/grass/lemon, and caramel to cocoa. The aromas of citrus, cream, and coconut were found to have significant negative correlations with bread, fat, and nuts, simultaneously (*p* < 0.05). In addition, grain was found to be closely related to several aroma sensory attributes in whiskey: grass, malt, spicy, and lemon. However, no single compound was identified as the source of the grain aroma in the GC-MS analyses above. Thus, the grain aroma in whiskeys is likely to result from a combination of compounds and aromas [6].

#### 3.3.2. Correlation Network Analysis between Volatile Substances and Sensory Evaluation

**Figure 5:**
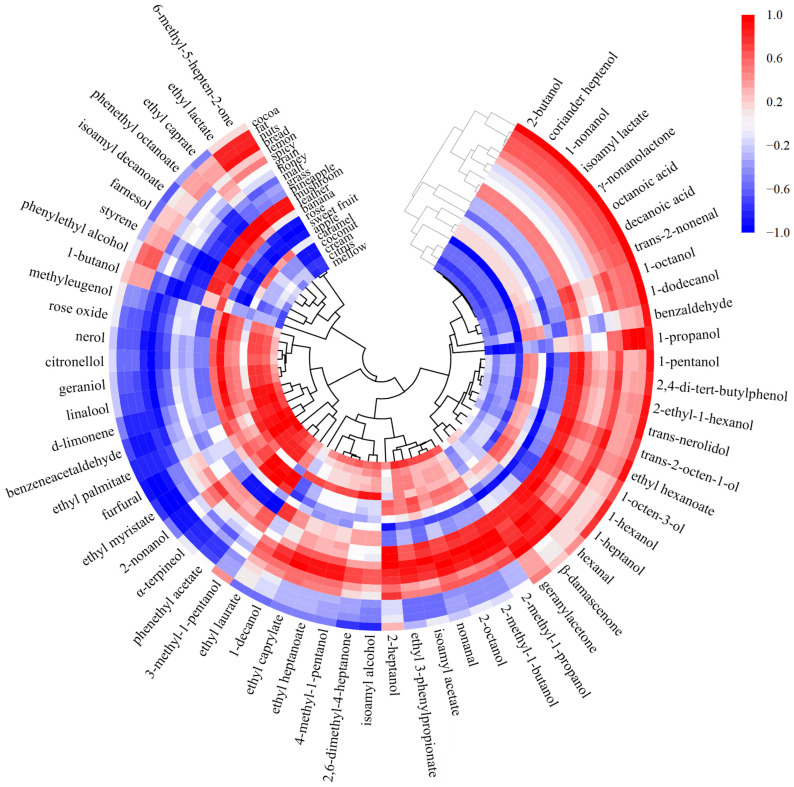
Correlation heat map between aroma sensory attributes and GC-MS analysis.

**Figure 6:**
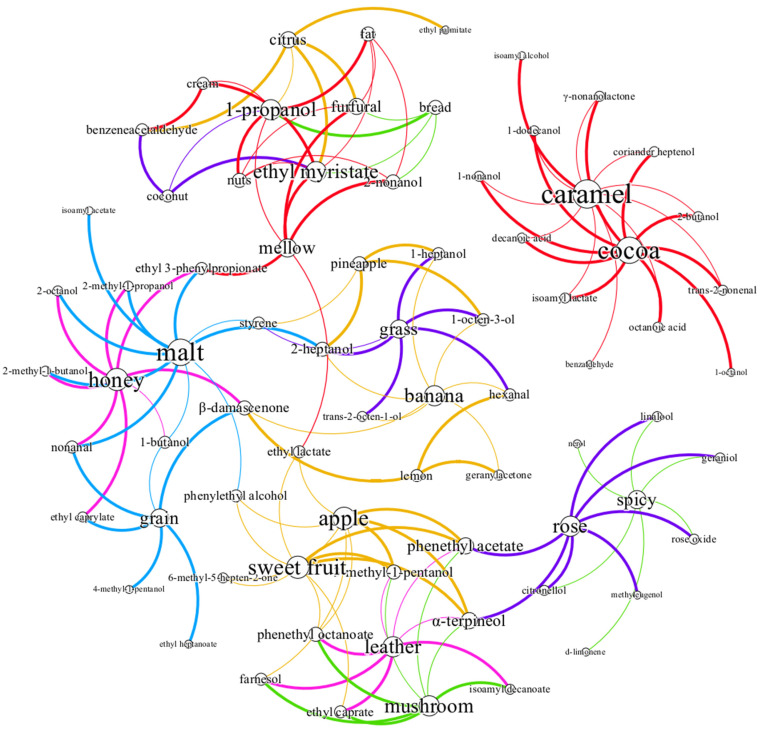
Correlation network diagram between aroma sensory attributes and GC-MS analysis. The size of the dot represents the strength of the correlations with other aroma sensory attributes or aroma substances, the thickness of the line represents the strength of the correlations between the two connected attributes or compounds, and the color of the line represents the correlations of different aroma sensory attributes.

A Pearson linear correlation analysis and heat map were applied, with a cluster analysis of the concentration of aroma substances in the GC-MS analysis and sensory evaluations (Figure 5). Data with a correlation coefficient greater than 0.9 were screened to establish the correlation network of whiskey aromas (Figure 6).

In Figure 5, the cocoa, fat, nuts, and bread aromas were grouped into a category, which was consistent with the correlation analysis of aroma sensory attributes above. These aromas were primarily associated with alcohols and esters, such as 1-propanol, 1-nonanol, coriander heptenol, ethyl myristate, ethyl palmitate, and γ-nonanolactone. Among these, γ-nonanolactone was shown to correlate negatively with the nut aroma [27] and positively with the butter aroma [28]. Cocoa was associated with many aroma compounds. *Trans*-2-nonenal, γ-nonanolactone, 1-nonanol, isoamyl lactate, 2-butanol, and 6-methyl-5-hepten-2-one were positively correlated with the cocoa aroma. γ-nonanolactone, 1-nonanol, isoamyl lactate, 2-butanol, and 6-methyl-5-hepten-2-one were exclusive to the highland barley whiskey. This may explain why the highland barley whiskey had a unique cocoa aroma in the sensory evaluation. Lemon, spicy, grain, honey, malt, grass and pineapple were grouped into one category. These aromas were mainly related to hexanal and geranylacetone with the grass aroma, β-damascenone with the honey aroma, and d-limonene and linalool with the citrus aroma. Among them, the OAV of β-damascenone was the highest and contributed the most (Table 1), being positively correlated with lemon, spicy, grain, honey, malt, grass, and pineapple.

The aromas of mushroom and leather were grouped into one category, and a strong positive correlation was found in the correlation analysis of the aroma sensory attributes above. 6-methyl-5-hepten-2-one, ethyl lactate, ethyl caprate, phenethyl octanoate, and farnesol were found to be positively correlated with the mushroom and leather aromas. Sorghum whiskey had the highest concentrations of these five compounds compared to the other whiskeys. Additionally, α-terpineol, 3-methyl-1-pentanol, and methyleugenol showed a strong negative correlation with the leather and mushroom aromas, and the concentrations of these three compounds were lowest in sorghum whiskey. These findings reflect the unique leather and mushroom aroma of sorghum whiskey.

Rose, sweet fruit, apple, caramel, coconut, cream, citrus, and mellow were consistent with the correlation analysis of aroma sensory attributes above (Section 2.3). These aromas were mainly related to terpenes and esters. Most of the esters such as ethyl myristate, ethyl palmitate, and phenethyl acetate (Table 1) exhibited a fruity and sweet aroma, and Sherman et al. also showed that esters usually have a sweet and fruity aroma [28]. Although present in small amounts, terpenes with a floral aroma, including linalool, nerol, geraniol, and α-terpineol, had a significant impact on the flavor of the whiskey (Table 1). Therefore, linalool was shown to be a positive contributor to the floral [29] and citrus aromas [30].

The aroma correlation network further illustrated the inner correlations between sensory attributes and aroma compounds. Figure 6 shows that the caramel aroma was correlated with several aroma substances, mainly alcohols. The most relevant aroma sensory attribute was the cocoa aroma. Only highland barley whiskey exhibited a distinct cocoa aroma. On the one hand, the substances positively correlated with cocoa (*trans*-2-nonenal, γ-nonanolactone, 1-nonanol, isoamyl lactate, and 2-butanol) in highland barley whiskey had higher concentrations than in the other three whiskeys. Secondly, the other three whiskeys had a more prominent caramel aroma. As mentioned earlier, caramel has a negative correlation with cocoa, and there may have been a masking effect between the aromas.

The substances related to the rose and spicy aromas were similar, mainly consisting of terpenes such as linalool and nerol with a citrus and floral aroma, and citronellol and geraniol with a rose aroma. Compounds associated with the nuts, bread, fat, coconut, cream, mellow, and citrus aromas were similar and identified mainly as ethyl myristate, phenylacetaldehyde, furfural, and 1-propanol. Among these compounds, phenylacetaldehyde had the highest OAV and made the largest contribution (Table 1), exhibiting a green, sweet, and floral aroma. In combination with Figure 4, a strong correlation was observed between these sensory attributes. Compounds related to honey, malt, and grain were similar. The same was true for pineapple, grass, and banana; mushroom and leather; and apple and sweet fruit. These were consistent with the research findings mentioned in Section 3.2.

**Table 1:** Aroma compounds identified in the four whiskeys.

No.	Compounds	RI	Concentration (mg/L) *				Odor Thresholds (mg/L)	Odour Activity Value (OAV)				Descriptor ^#^
			Barley Whiskey	Wheat Whiskey	Sorghum Whiskey	Highland Barley Whiskey		Barley Whiskey	Wheat Whiskey	Sorghum Whiskey	Highland Barley Whiskey	
	**Alcohols**											
1	2-Butanol	1038.4	nf	nf	nf	3.6 ± 0.034 a	50 ^ζ^	-	-	-	<1	fruity, sweet, apricot
2	1-Propanol	1053.0	41.379 ± 4.75 a	40.29 ± 9.833 a	45.26 ± 1.818 a	49.423 ± 0.997 a	53.952 ^δ^	<1	<1	<1	<1	sweet, fruity, apple
3	2-Methyl-1-propanol	1105.7	35.613 ± 9.764 a	18.207 ± 3.855 a	20.472 ± 0.605 a	24.373 ± 0.254 a	28.3 ^δ^	1.26	<1	<1	<1	ethereal, winey
4	1-Butanol	1155.1	0.45 ± 0.021 c	1.081 ± 0.056 a	0.957 ± 0.021 b	0.96 ± 0.01 b	2.73 ^γ^	<1	<1	<1	<1	oily, sweet, balsamic
5	2-Methyl-1-butanol	1208.1	7.903 ± 0.61 a	7.131 ± 0.469 a	7.179 ± 0.208 a	7.303 ± 0.149 a	24 ^α^	<1	<1	<1	<1	fatty, leathery, cocoa
6	Isoamyl alcohol	1208.4	281.389 ± 35.864 a	251.732 ± 15.31 a	269.861 ± 8.853 a	231.586 ± 4.885 a	56.1 ^η^	5.02	4.49	4.81	4.13	whiskey, fruity, banana
7	1-Pentanol	1247.4	0.31 ± 0.016 b	0.202 ± 0.008 c	0.18 ± 0.005 c	0.432 ± 0.001 a	64.43 ^ζ^	<1	<1	<1	<1	oily, sweet, balsamic
8	4-Methyl-1-pentanol	1307.1	0.039 ± 0.002 a	nf	0.019 ± 0.001 b	nf	nf	-	-	-	-	nutty
9	2-Heptanol	1311.6	0.014 ± 0.002 a	nf	nf	0.009 ± 0.00 b	1.434 ^ε^	<1	-	-	<1	mushroom, oily, fatty
10	3-Methyl-1-pentanol	1321.6	0.035 ± 0.006 a	0.037 ± 0.001 a	nf	0.041 ± 0.001 a	1 ^ζ^	<1	<1	-	<1	winey, cocoa, green, fruity
11	1-Hexanol	1348.3	0.194 ± 0.026 b	0.118 ± 0.003 c	0.068 ± 0.003 d	0.279 ± 0.00 a	0.537 ^θ^	<1	<1	<1	<1	oily, fruity, sweet, green
12	2-Octanol	1414.9	0.011 ± 0.002 a	nf	nf	0.002 ± 0.00 b	0.0715 ^ζ^	<1	-	-	<1	spicy, green, woody
13	1-Octen-3-ol	1442.9	0.113 ± 0.025 a	0.016 ± 0.004 b	0.027 ± 0.001 b	0.145 ± 0.002 a	0.006 ^θ^	18.87	2.62	4.44	24.24	earthy, green, oily
14	1-Heptanol	1447.7	0.079 ± 0.01 a	0.057 ± 0.003 b	0.053 ± 0.001 b	0.09 ± 0.001 a	26.6 ^δ^	<1	<1	<1	<1	leafy, green, fruity, apple
15	Coriander heptenol	1453.5	nf	nf	nf	0.053 ± 0.004 a	2 ^ζ^	-	-	-	<1	sweet, oily, green
16	2-Ethyl-1-hexanol	1477.5	0.012 ± 0.003 b	nf	nf	0.026 ± 0.001 a	24.623 ^ε^	<1	-	-	<1	citrus, floral, oily, sweet
17	2-Nonanol	1502.8	0.018 ± 0.004 a	0.009 ± 0.001 b	nf	nf	0.058 ^ζ^	<1	<1	-	-	waxy, green, creamy
18	1-Octanol	1544.0	0.033 ± 0.007 b	0.014 ± 0.001 c	0.02 ± 0.001 c	0.076 ± 0.002 a	1.1 ^δ^	<1	<1	<1	<1	waxy, green, citrus, floral
19	*Trans*-2-octen-1-ol	1604.4	0.04 ± 0.011 a	0.011 ± 0.00 b	0.02 ± 0.002 b	0.054 ± 0.001 a	0.02 ^ζ^	1.99	<1	<1	2.69	green, citrus, fatty
20	1-Nonanol	1648.7	nf	nf	nf	0.148 ± 0.006 a	0.0455 ^ζ^	-	-	-	3.25	fatty, floral, rose, orange
21	1-Decanol	1755.6	0.025 ± 0.007 a	nf	0.021 ± 0.001 a	nf	0.5 ^ζ^	<1	-	<1	-	fatty, waxy, floral, orange
22	Phenylethyl alcohol	1916.6	5.755 ± 2.134 a	7.4 ± 0.16 a	9.158 ± 0.603 a	7.164 ± 1.098 a	2.6 ^η^	2.21	2.85	3.52	2.76	sweet, floral, rose
23	1-Dodecanol	1958.5	0.026 ± 0.009 b	0.013 ± 0.003 b	0.014 ± 0.002 b	0.072 ± 0.008 a	1.001 ^ζ^	<1	<1	<1	<1	earthy, soapy, waxy, fatty
24	Farnesol	2299.6	0.016 ± 0.008 b	0.027 ± 0.003 b	0.085 ± 0.021 a	nf	5 ^ζ^	<1	<1	<1	-	fresh, sweet, floral
	**Esters**											
25	Isoamyl acetate	1117.0	0.918 ± 0.498 a	0.277 ± 0.026 a	0.157 ± 0.02 a	0.362 ± 0.001 a	0.245 ^β^	3.75	1.13	<1	1.48	sweet, banana, fruity
26	Ethyl hexanoate	1226.2	0.056 ± 0.012 b	0.013 ± 0.001 c	0.023 ± 0.002 c	0.096 ± 0.005 a	0.0553 ^θ^	1.02	<1	<1	1.74	sweet, fruity, pineapple
27	Ethyl heptanoate	1327.9	0.002 ± 0.00 a	nf	0.001 ± 0.00 b	nf	13.2 ^γ^	<1	-	<1	-	fruity, pineapple, winey
28	Ethyl lactate	1343.7	0.571 ± 0.06 c	0.591 ± 0.092 c	1.168 ± 0.062 a	0.861 ± 0.088 b	128.084 ^δ^	<1	<1	<1	<1	sweet, fruity, acidic
29	Ethyl caprylate	1431.0	0.102 ± 0.02 a	0.058 ± 0.004 b	0.081 ± 0.004 ab	0.061 ± 0.003 b	0.147 ^η^	<1	<1	<1	<1	fruity, winey, waxy, sweet
30	Isoamyl lactate	1562.4	nf	nf	nf	0.258 ± 0.026 a	nf	-	-	-	-	fruity, creamy, nutty
31	Ethyl caprate	1630.0	0.062 ± 0.01 b	0.053 ± 0.009 b	0.093 ± 0.003 a	0.05 ± 0.003 b	1.12 ^γ^	<1	<1	<1	<1	sweet, waxy, fruity, apple
32	Phenethyl acetate	1824.8	0.575 ± 0.195 a	0.647 ± 0.027 a	0.342 ± 0.009 a	0.518 ± 0.026 a	0.908 ^δ^	<1	<1	<1	<1	floral, rose, sweet, honey
33	Ethyl laurate	1847.1	0.583 ± 0.052 b	nf	0.874 ± 0.142 a	nf	0.4 ^ε^	1.46	-	2.18	-	sweet, waxy, floral, soapy
34	Isoamyl decanoate	1861.5	0.018 ± 0.003 bc	0.027 ± 0.003 b	0.048 ± 0.009 a	0.009 ± 0.001 c	>5 ^ζ^	<1	<1	<1	<1	waxy, banana, fruity, sweet
35	Ethyl 3-phenylpropionate	1893.4	0.002 ± 0.001 a	nf	nf	nf	0.013 ^θ^	<1	-	-	-	rose, honey, fruity
36	γ-Nonanolactone	2036.6	nf	nf	nf	0.385 ± 0.056 a	0.0097 ^θ^	-	-	-	39.67	coconut, creamy, waxy
37	Ethyl myristate	2038.7	0.482 ± 0.09 a	0.459 ± 0.064 a	0.23 ± 0.041 b	0.13 ± 0.021 b	46.606 ^δ^	<1	<1	<1	<1	sweet, waxy
38	Ethyl palmitate	2218.3	1.221 ± 0.477 a	1.522 ± 0.177 a	1.097 ± 0.171 a	0.669 ± 0.082 a	39.299 ^δ^	<1	<1	<1	<1	waxy, fruity, creamy
39	Phenethyl octanoate	2332.8	0.04 ± 0.01 a	0.052 ± 0.006 a	0.07 ± 0.015 a	0.043 ± 0.005 a	10 ^ζ^	<1	<1	<1	<1	sweet, waxy, green, cocoa
	**Aldehydes**											
40	Hexanal	1083.9	0.059 ± 0.001 a	nf	0.024 ± 0.002 c	0.049 ± 0.001 b	0.0255 ^θ^	2.33	-	<1	1.93	green, fatty, fruity
41	Nonanal	1395.7	0.014 ± 0.003 a	0.004 ± 0.001 b	0.006 ± 0.001 b	0.006 ± 0.00 b	0.122 ^δ^	<1	<1	<1	<1	waxy, aldehydic, citrus
42	Furfural	1466.2	4.833 ± 1.066 a	4.816 ± 0.203 a	3.382 ± 0.204 ab	1.927 ± 0.103 b	44.029 ^δ^	<1	<1	<1	<1	sweet, woody, bread
43	Benzaldehyde	1525.9	0.204 ± 0.05 bc	0.301 ± 0.029 b	0.161 ± 0.01 c	0.455 ± 0.008 a	4.203 ^δ^	<1	<1	<1	<1	fruity, powdery, nutty
44	*Trans*-2-nonenal	1531.2	0.027 ± 0.001 b	0.028 ± 0.00 b	0.027 ± 0.00 b	0.105 ± 0.002 a	0.0006 ^η^	45.80	46.28	44.22	175.20	fatty, green, cucumber
45	Phenylacetaldehyde	1649.0	0.33 ± 0.085 ab	0.47 ± 0.032 a	0.229 ± 0.006 bc	0.162 ± 0.009 c	0.111 ^β^	2.97	4.23	2.07	1.46	green, sweet, floral, honey
	**Terpenes**											
46	D-limonene	1181.5	0.027 ± 0.003 b	0.072 ± 0.015 a	nf	nf	nf	-	-	-	-	citrus, orange, sweet
47	Styrene	1253.0	0.003 ± 0.002 a	0.006 ± 0.00 a	0.007 ± 0.001 a	0.004 ± 0.00 a	0.125 ^ζ^	<1	<1	<1	<1	sweet, balsamic, floral
48	Linalool	1534.0	0.059 ± 0.023 b	0.148 ± 0.016 a	0.024 ± 0.001 b	0.05 ± 0.001 b	0.023 ^β^	2.55	6.45	1.06	2.18	citrus, floral, sweet, rose
49	α-terpineol	1694.6	0.034 ± 0.003 a	0.035 ± 0.001 a	0.029 ± 0.00 b	0.032 ± 0.00 ab	1.96 ^δ^	<1	<1	<1	<1	floral, terpenic
50	Citronellol	1760.8	0.779 ± 0.391 bc	4.103 ± 0.263 a	0.224 ± 0.008 c	1.187 ± 0.117 b	0.1 ^ζ^	7.79	41.03	2.24	11.87	floral, rosy, sweet, citrus
51	β-Damascenone	1831.0	2.431 ± 0.752 a	0.021 ± 0.00 c	1.032 ± 0.02 bc	1.592 ± 0.065 ab	0.00012 ^θ^	20,260	171	8600	13,270	apple, rose, honey, tobacco
52	Nerol	1797.9	0.065 ± 0.021 b	0.315 ± 0.011 a	0.02 ± 0.001 c	0.07 ± 0.007 b	0.5 ^ζ^	<1	<1	<1	<1	citrus, floral, green, sweet
53	Geraniol	1843.3	0.339 ± 0.129 b	1.416 ± 0.048 a	nf	0.286 ± 0.044 b	0.2 ^ζ^	1.69	7.08	-	1.43	floral, sweet, rosey, fruity
54	*Trans*-nerolidol	2028.4	0.059 ± 0.022 a	0.044 ± 0.008 a	0.043 ± 0.007 a	0.078 ± 0.007 a	1 ^ζ^	<1	<1	<1	<1	floral, green, citrus, woody
	**Others**											
55	2,6-Dimethyl-4-heptanone	1165.2	0.336 ± 0.054 a	0.247 ± 0.025 a	0.309 ± 0.035 a	0.207 ± 0.145 a	nf	-	-	-	-	green, fruity, pineapple, banana
56	6-Methyl-5-hepten-2-one	1337.1	0.036 ± 0.004 b	0.035 ± 0.006 b	0.073 ± 0.004 b	0.053 ± 0.004 a	1.008 ^ζ^	<1	<1	<1	<1	citrus, green, lemongrass, apple
57	Rose oxide	1351.5	0.019 ± 0.005 bc	0.061 ± 0.008 a	0.003 ± 0.00 c	0.024 ± 0.001 b	nf	-	-	-	-	green, rose, spicy
58	Geranylacetone	1858.1	0.01 ± 0.004 a	0.005 ± 0.001 a	0.008 ± 0.001 a	0.01 ± 0.001 a	nf	-	-	-	-	rose, leafy, floral, green
59	Methyleugenol	2010.4	0.025 ± 0.01 bc	0.083 ± 0.006 a	0.007 ± 0.001 c	0.044 ± 0.005 b	10 ^ζ^	<1	<1	<1	<1	spicy, cinnamon, clove
60	Octanoic acid	2046.6	nf	nf	nf	1.815 ± 0.144 a	2.7 ^γ^	-	-	-	<1	fatty, waxy, oily
61	Decanoic acid	2233.8	nf	nf	nf	1.101 ± 0.126 a	2.8 ^α^	-	-	-	<1	sour, fatty, citrus
62	2,4-Di-*tert*-butylphenol	2262.5	0.01 ± 0.004 b	nf	nf	0.018 ± 0.001 a	0.373 ^ε^	<1	-	-	<1	nf

* Different letters indicate significant differences in the same line (*p* < 0.05); “nf” means not found; ^#^ descriptor was quoted from http://www.thegoodscentscompany.com/allproc-1.html (accessed on 8 October, 2023); ^α^ odor thresholds were determined in 40% ethanol/water, quoted from [31]; ^β^ odor thresholds were determined in 60% ethanol/water, quoted from [32]; ^γ^ odor thresholds were determined in 46% ethanol/water, quoted from [33]; ^δ^ odor thresholds were determined in 46% ethanol/water, quoted from [34]; ^ε^ odor thresholds were determined in 53% ethanol/water, quoted from [35]; ^ζ^ quoted from [36]; ^η^ odor thresholds were determined in 40% ethanol/water, quoted from [37]; ^θ^ odor thresholds were determined in 46% ethanol/water, quoted from [15].

## 4. Conclusions

Barley whiskey had the highest number of aroma substances (55) and a relatively higher content of volatiles, followed by highland barley whiskey (54). Wheat whiskey and sorghum whiskey contained similar aroma compounds and concentrations. All four whiskeys were found to be predominantly composed of alcohols. In comparison, sorghum whiskey exhibited a high concentration of esters, whereas wheat whiskey was rich in terpenes. The OAVs of isoamyl alcohol (231.59~281.39 mg/L), phenylethyl alcohol (5.755~9.158 mg/L), citronellol (0.224~4.103 mg/L), β-damascenone (0.021~2.431 mg/L), geraniol (0.286~1.416 mg/L), isoamyl acetate (0.157~0.918 mg/L), phenylacetaldehyde (0.162~0.470 mg/L), linalool (0.024~0.148 mg/L), 1-octen-3-ol (0.016~0.145 mg/L), *trans*-2-nonenal (0.027~0.105 mg/L), and *trans*-2-octen-1-ol (0.011~0.054 mg/L) were all higher than 1, which demonstrated that all of these aroma compounds are significant in whiskey.

The fruity (citrus, pineapple, sweet fruit, apple), floral (coconut, grass, rose), and grain (malt and grain) aromas in barley whiskey and wheat whiskey were more prominent, which was attributed to the presence of esters and terpenes in the whiskey. Compared with the previous two, sorghum whiskey and highland barley whiskey were similar in their pineapple, citrus, grass, malt, grain, honey, and mellow aromas, while the spicy and bread aromas scored higher. Sorghum whiskey and highland barley whiskey were more characteristic. Sorghum whiskey has a specific leather and mushroom aroma. By means of correlation analysis, this aroma may be attributed to 6-methyl-5-hepten-2-one, ethyl lactate, ethyl caprate, phenethyl octanoate, farnesol, α-terpineol, 3-methyl-1-pentanol, and methyleugenol. Highland barley whiskey had the greatest number of unique aroma compounds (seven). It showed a unique cocoa aroma, which may be related to concentrations of *trans*-2-nonenal, γ-nonanolactone, 1-nonanol, isoamyl lactate, 2-butanol, and 6-methyl-5-hepten-2-one, which were the exclusive aroma components of highland barley whiskey. The other three whiskeys had a more prominent caramel aroma, which may have had a masking effect on the cocoa aroma.

## Data Availability

The original contributions presented in the study are included in the article, and further inquiries can be directed to the corresponding author.
